# Unilateral Acute Idiopathic Optic Neuritis With Superior Altitudinal Visual Field Defect as a Presenting Feature

**DOI:** 10.7759/cureus.17011

**Published:** 2021-08-08

**Authors:** Ali Anwar Abro, Aysha Falamarzi, Mohamed Yusuf Shaikh

**Affiliations:** 1 Eye and Laser Centre, Royal Medical Services / Bahrain Defense Force Hospital, East Riffaa, BHR

**Keywords:** acute optic neuritis, idiopathic optic neuritis, altitudinal visual field defect, presentation of optic neuritis, atypical optic neuritis

## Abstract

Patients with acute optic neuritis typically present with acute loss of vision. We describe a case of a young lady of 25 years of age with blurring of vision in the upper visual field of the right eye with otherwise intact visual acuity as the only presenting symptom. Although altitudinal visual field defect is not unknown to be associated with acute optic neuritis, it is generally considered a relatively uncommon occurrence. Our case illustrates an unusually unique occurrence of upper altitudinal visual field defect in association with unaffected visual acuity as the sole presenting symptom of acute idiopathic unilateral optic neuritis. When an altitudinal visual field defect is a presenting feature, besides the usual vascular and compressive causes, optic neuritis should be remembered in the list of differential diagnoses.

## Introduction

Inflammatory optic neuropathy, or optic neuritis (ON), is the most common cause of optic nerve injury in young adults. ON has multiple etiologies, including demyelinating, infectious, and autoimmune causes. It can also occur independently as idiopathic optic neuritis.

A variety of visual field defects can be found in optic neuritis. The Optic Neuritis Treatment Trial (ONTT) identified different types of visual field defects at the baseline in the affected eyes. Diffuse visual field loss was present in 66% of the affected eyes and central field loss in 27%. Altitudinal visual field abnormality which is classically believed to be highly characteristic of the ischemic optic neuropathy occurred in 8% of the affected eyes at the baseline in ONTT [1]. 

Acute onset altitudinal visual field defect, which involves loss of visual sensation in the horizontal half of the visual field, often vascular in origin, can rarely be caused by compressive neuropathy due to a tumor or aneurysm [2]. Acute optic neuritis is generally considered a relatively uncommon cause of the altitudinal field defect.

Patients with optic neuritis typically present with acute unilateral loss of vision usually in association with impaired color vision. We describe a case of a young lady of 25 years of age who presented with acute onset of blurred vision in the upper visual field with no subjective changes either in visual acuity or color vision. Humphry’s visual field plot confirmed the presence of superior altitudinal scotoma in the affected eye. The local and systemic investigations suggested the idiopathic nature of acute optic neuritis. Our case therefore aims to illustrate an unusual scenario of subjective blurring in the superior visual field from altitudinal scotoma as a presenting feature of acute unilateral idiopathic optic neuritis.

## Case presentation

A 25-year-old Bahraini female presented to the eye clinic with blurring of vision in the upper field of her right eye of four days duration. There was no history of preceding febrile illness, immunization, trauma, or any past systemic or neurological disease or any medications. Her past ocular history included laser correction in both the eyes for a mild myopic refractive error four years ago. There was no significant family history. Her visual acuity on presentation was 6/6 in each eye. Ishihara test revealed a mildly impaired color vision in the right eye and fully normal color vision in the left eye. Ocular movements showed a full range but were associated with some pain on supraduction of the right eye.

**Figure 1:**
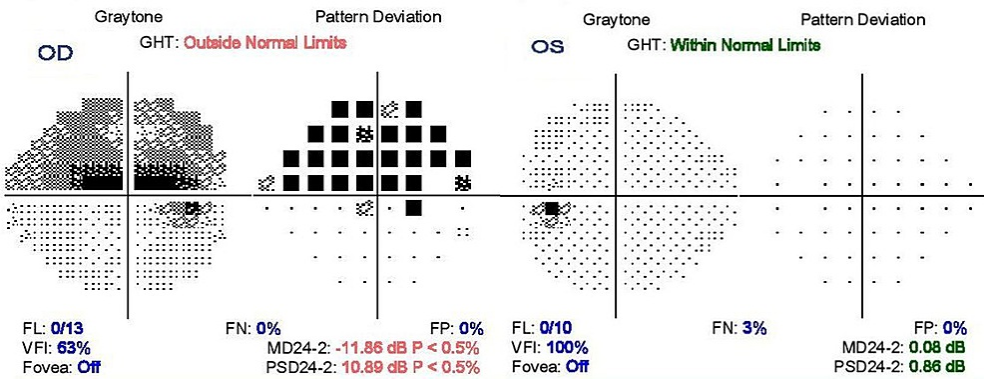
Humphrey 24-2 visual field plot at presentation showing superior altitudinal visual field defect in the right eye (OD) and normal visual field plot in the left eye (OS).

Anterior segment and adnexal examinations were unremarkable. A relative afferent pupillary defect grade two was noted in the right eye. Fundus examination revealed normal-looking optic disc, retinal and vasculature details. Her visual field plot, 24-2 on Humphrey’s field analyzer, revealed in the right eye a superior altitudinal visual field defect (Figure 1). The fellow eye showed a normal field plot. Optical coherence tomography and B scan ultrasonography of the optic discs revealed no abnormalities. 

Systemic evaluation including full neurological evaluation and ENT status were within normal limits. Brain and orbit MRI scans were unremarkable. Blood investigations including full blood count, renal function tests, erythrocyte sedimentation rate, C-reactive protein, serum folate and B 12 levels were all also within normal. Serum autoimmune markers such as anti-cardiolipin antibodies, antinuclear antibody (ANA) screen and double-stranded DNA were also within normal range. Serological infective screening that included TP-antibody for syphilis, anti-hepatitis C virus (HCV), HIV AB/AG combo and hepatitis B surface antigen were all non-reactive. Serum anti-aquapore-4 antibody test also returned negative. Electrophysiological study of visual-evoked cortical potential (VEP) revealed delayed latencies suggestive of optic neuritis. 

**Figure 2:**
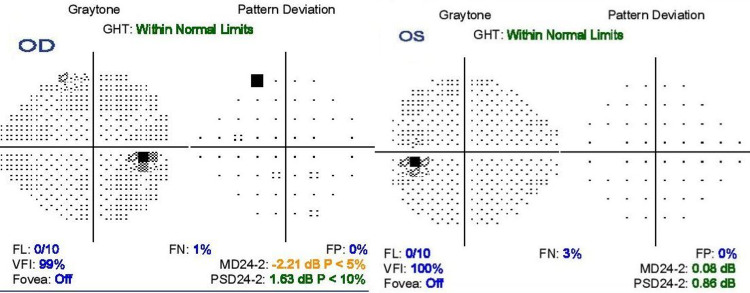
Humphrey 24-2 visual field plot post-treatment on 11th day of presentation showing resolved superior altitudinal visual field defect in the right eye (OD) and normal visual field plot in the left eye (OS).

Two days after the presentation, her visual acuity deteriorated to 6/15 at which point she was started on a five-day course of high dose intravenous methylprednisolone 1 gm daily under the care of a neurophysician. On her review four days after the completion of the treatment, that is on the 11th day from the initial presentation, she was noted to have total recovery of the visual acuity, full restoration of the color vision on Ishihara’s testing and complete disappearance of the altitudinal field defect on Humphrey’s perimetry (Figure 2).

## Discussion

Inflammatory optic neuropathy, or optic neuritis (ON), which can be defined as inflammation of the optic nerve due to various causes, is the most common optic neuropathy under 50 years among general ophthalmic practice [3]. Optic neuritis has multiple etiologies, including demyelinating, infectious, and autoimmune causes. Isolated optic neuritis not associated with any specific neurological or systemic disease is labeled as idiopathic optic neuritis. However, cases presenting as idiopathic optic neuritis may be the initial presentation in 20% of multiple sclerosis patients [4]. 

Altitudinal visual field defect in optic neuritis, though not completely unknown, is a relatively uncommon feature of optic neuritis. The patients included in the Optic Neuritis Treatment Trial (ONTT) presented with various types of visual field defects in the affected eye, with two-thirds showing diffuse defects and one-third with localized field defects. Keltner et al. [1] observed that only 8% of the participants from the ONTT presented with altitudinal field defect. Altitudinal defects have been reported both in superior and inferior half of the fields in optic neuritis.

It is suggested that either the inflammation of the optic nerve itself or the secondary perfusion defect caused by the inflammation of the optic nerve may lead to the altitudinal field defect in optic neuritis [5]. Chin and Ismail described a case of multiple sclerosis-associated optic neuritis in a 17-year old girl presenting with severe reduction in visual acuity in association with inferior altitudinal defect [6]. Our case is different in a way that the superior altitudinal defect was the presenting subjective complaint with no changes in the visual acuity. Kale et al. [4] suggested that typically the presence of altitudinal defect should warrant a consideration of other non-inflammatory differential diagnoses. Its presence should therefore alert the clinician to look for more classic causes of vascular or compressive in nature. An interesting occurrence of acute onset altitudinal field defect has been described as a sole presenting feature of compressive lesion of intracanalicular meningioma [7]. Clinical profile of the patient along with pain on upgaze and normal looking discs with reduced color vision and prolonged latencies on VEP all pointed towards an inflammatory cause in our patient. There was no radiological evidence of any abnormality including any demyelinating lesions on magnetic resonance imaging of brain and orbit. Clinical and laboratory investigations excluded other causes of optic neuritis from infective, autoimmune and adjacent sinus disease. Optic neuritis associated with neuromyelitis optica (NMO) remained an important differential diagnosis as it is known to have higher incidence of non-central scotoma and altitudinal defects [5]. There was no clinical or serological evidence for NMO in our case. Our case of idiopathic optic neuritis presenting with upper visual field defect with intact visual acuity is a scenario not described in the literature to the best of our knowledge. There is a possibility that the current etiological diagnosis of idiopathic origin may alter in future on further follow up. 

A number of randomized, controlled, double-blind trials of corticosteroid treatment of optic neuritis were evaluated in a meta-analysis in 2012. One randomized and controlled trial, the Optic Neuritis Treatment Trial, had a major effect on the current standard of treatment. In this trial, oral prednisone treatment at a dose of 1 mg/kg body weight/day for 14 days was compared with intravenous methylprednisolone treatment at 1000 mg/day for three days followed by oral prednisolone (1 mg/kg BW) for 11 days, and with placebo treatment. Treatment with intravenous methylprednisolone, which was not blinded, led to a more rapid recovery of vision, but the final outcome with respect to visual acuity, fields, and perception of contrast and color was no better than with oral prednisone alone, or indeed with placebo. Similar results were found in earlier and later studies as well; thus, it was concluded in the meta-analysis of 2012 that faster recovery is the sole benefit of steroid treatment. Among the patients in the Optic Neuritis Treatment Trial who were treated only with low-dose oral prednisolone, early recurrences within six months were twice as common as in the placebo group. Since the publication of these findings, low-dose oral prednisolone alone has been considered to be contraindicated for patients with typical optic neuritis [8].

The patients in the Optic Neuritis Treatment Trial who received intravenous methylprednisolone for three days all also received oral prednisolone over the ensuing 11 days. It is unclear whether this is necessary, and the guidelines leave the question open. Some authorities do not give oral prednisolone after intravenous methylprednisolone. Some current neurological and ophthalmological guidelines advocate treatment of optic neuritis with methylprednisolone at a dose of 500-1000 mg/day for three to five days [8].

For patients with ON whose brain lesions on magnetic resonance imaging indicate a high risk of developing clinically definite multiple sclerosis, treatment with immunomodulators (eg, interferon beta-1a, interferon beta-1b, glatiramer acetate) may be considered. Intravenous immunoglobulin treatment of acute ON has been shown to have no beneficial effect [8].

Recovery of visual function in acute optic neuritis is known to occur within the first month of the onset. In the ONTT, 79% of the participants had started to improve by three weeks and 93% by five weeks [9]. In our patient, four days after the completion of the course of a high dose of intravenous methylprednisolone, altitudinal field defect disappeared completely. 

In summary, we described a case of acute idiopathic unilateral optic neuritis with the subjective complaint of upper altitudinal visual field defect as a sole presenting feature in the presence of normal visual acuity. 

## Conclusions

Optic neuritis is a multifaceted disease that may have atypical clinical presentations. The clinician should be familiar with atypical presentations which should prompt other differential diagnoses without ruling out the diagnosis of acute optic neuritis. If atypical features are present, urgent further investigations are indicated to exclude the differential diagnoses. Our case of acute idiopathic unilateral optic neuritis had an unusual presenting symptom of upper visual field blurring in the presence of unaffected visual acuity. When an altitudinal visual field defect is a presenting feature, besides the usual vascular and compressive causes, optic neuritis should be remembered in the list of differential diagnoses. Further imaging to exclude other intracranial or intraorbital pathologies is warranted. Brain MRI is important diagnostically and prognostically in monitoring the future development and progression of demyelinating disease. Last but not the least, in the case of young healthy female patients, diagnostic work up and co-management with a neuromedical team would ensure an optimal work up and the appropriate future follow up necessary for such patients.
